# Clinical Chemistry Reference Intervals for Health Assessment in Wild Adult Harbour Seals

**DOI:** 10.3390/ani15233429

**Published:** 2025-11-27

**Authors:** Ailsa J. Hall, Debbie J. F. Russell, Paul M. Thompson, Ryan Milne, Simon E. Moss, Holly C. Armstrong, Joanna L. Kershaw

**Affiliations:** 1 Sea Mammal Research Unit, Scottish Oceans Institute, University of St. Andrews, St. Andrews KY16 8LB, UK; dr60@st-andrews.ac.uk (D.J.F.R.); rm610@st-andrews.ac.uk (R.M.); sem6@st-andrews.ac.uk (S.E.M.); ha54@st-andrews.ac.uk (H.C.A.); jk49@st-andrews.ac.uk (J.L.K.); 2 Lighthouse Field Station, School of Biological Sciences, University of Aberdeen, George St., Aberdeen IV11 8YJ, UK; paul.thompson@abdn.ac.uk

**Keywords:** biochemistry, blood parameters, pinnipeds, disease

## Abstract

Reference intervals for clinical chemistry blood parameters are valuable for both individual diagnostics for animals in managed or veterinary care, and for evaluating wild population health. However, for marine mammals obtaining sufficient data from suitable groups or populations is logistically difficult. Here, we have assembled a large dataset of clinical chemistry results from free-living adult UK harbour seals (*Phoca vitulina*), analysed in the same commercial laboratory. We applied an open-source algorithm (available as the R package *refineR*, R version 4.5.2, *refineR* version 2.0.0) to produce robust reference intervals from these Real-World Data. This novel approach resulted in the generation of 95% reference intervals with 90% confidence bounds for 18 key chemistry parameters indicative of a range of physiological processes including, inflammation, nutritional status, kidney function and liver function. Reference intervals were also generated for triiodothyronine, the active thyroid hormone important in the regulation of metabolism. These intervals will provide critical baseline data for the assessment of harbour seal health as, to our knowledge, this is the largest dataset on which clinical chemistry reference intervals from wild-caught adult harbour seals have been based.

## Simple Summary

We produced robust clinical chemistry reference intervals for wild adult harbour seals. A large dataset of 317 serum samples was analysed for 18 clinical chemistry parameters and one thyroid hormone. Reference intervals were generated using an approach developed for the medical field that makes no assumptions about underlying health conditions and is suitable for data from both pathological and non-pathological subjects. The resulting reference intervals can be used diagnostically and prognostically for assessing the health of harbour seals in a variety of settings.

## 1. Introduction

Clinical chemistry parameters are used to assess health and diagnose disease in animals across a wide range of settings, from individuals through veterinary practice [1] to population-level health assessment to inform conservation and management decisions [2]. Defined as the central 95% of a distribution of data from individuals that are selected randomly from the population [3], it is an approach long established in human and veterinary medicine [4]. However, establishing robust baseline reference intervals for comparison, particularly in wild marine mammal species, is challenging as certain criteria must be met before reference intervals are sufficiently reliable. The large sample size required can be a stumbling block since logistical handling and sampling constraints in marine mammals mean that blood samples often cannot be collected, and for those where sampling is possible, only small sample sizes are available from limited regions or seasons [5,6]. The selection of an appropriate reference group or population is important, and selection criteria are dictated by the ultimate use for the data. For a “direct” method, individuals who meet certain inclusion (and exclusion) criteria are selected such that individuals are apparently healthy [7]. However, the utility and application of this approach have been widely debated in relation to human health [8] as recruiting sufficient overtly healthy subjects is not possible in many situations. Testing the recommendation that reference values should be derived from healthy individuals found that “healthy” people are such a minority in a population that using this as a means to evaluate health was almost impossible [9]. The International Federation of Clinical Chemistry and Laboratory Medicine (IFCC) then recommended that for any given reference interval, its use and method of selecting the reference individuals should be defined [3,10]. Similar guidance has also been published for deriving reference intervals for use in a veterinary setting for companion animals and livestock [11,12]. Thus, “indirect” methods were subsequently developed [11,13], in which data from routine measurements from entire populations (so-called Real-World Data, RWD) were analysed [14,15]. These approaches assume that the dataset will include individuals with disease, i.e., test results that are pathological as well as non-pathological, under the assumption that most results within the dataset are non-pathological. In this approach, defining healthy individuals is therefore not necessary. This is thus entirely suitable for the analysis of data and the generation of clinical chemistry reference intervals for wild, free-living marine mammals, provided a large enough sample size is available.

In addition, how the intervals should be calculated [16] and how wide they should be [17], have been the subject of discussion [10], with the consensus within the IFCC that using the central 95% of the distribution with 90% confidence intervals around the upper and lower bounds would provide the most reliable indicator of uncertainty. The use of parametric methods is not recommended due to the non-Gaussian distribution of most parameters [18].

Wild capture, sample, and release studies involving marine mammals have now been carried out for many species over relatively long time periods [19]. Some studies also involved collecting blood samples and, using standardised methods, determined baseline reference intervals [6,20]. A good example of this is the bottlenose dolphin (*Tursiops truncatus*), where health assessments among free-living populations across the US have been carried out over the past 30 years [2,21], establishing haematological and clinical chemistry reference intervals that have then been critical in assessing the health of individual animals. These health metrics have then been crucially important in determining anthropogenic impacts [22,23,24] and providing early warning signs of population-level perturbations [25,26,27].

Harbour seals (*Phoca vitulina*) have been the subject of a variety of capture, sample, and release research projects to answer different questions relating to their ecology and biology, and to determine the impact of anthropogenic stressors [28,29,30,31,32,33,34,35]. These include animals at sites from all around the Scottish coast and in the Wash, which has the largest harbour seal population in England [36]. During these projects, blood samples were collected and analysed for a standard suite of clinical chemistry parameters, and concentrations of the active thyroid hormone, triiodothyronine, were quantified. This amounts to a 27-year dataset of 317 individuals, which can now be used to establish a set of robust reference intervals, using the same approach for the US bottlenose dolphins [21] through an RWD analytical framework. Although reference intervals for wild adult harbour seal clinical chemistry parameters have been published [37], those data were from the Pacific subspecies (*Phoca vitulina richardii*, *n* = 57). Here, we report the largest dataset for *Phoca vitulina vitulina* to date, and intervals have been generated using the IFCC-recommended statistical approach through a newly developed RWD framework [38]. The results provide valuable baseline data for evaluating the health of harbour seal populations that are currently displaying very different population trajectories, from declining to stable or increasing [36,39].

## 2. Materials and Methods

### 2.1. Sample Collection

Methods for the capture and release of harbour seals have been previously published [34,40], with all studies being carried out under licences from the Home Office in compliance with the Animal (Scientific Procedures) Act 1986 (PPL numbers 60/2069, 60/2589, 60/3303, 60/4009 70/7806, PF84B63DE and PP0562940), the Conservation of Seals Act 1970 and the Marine (Scotland) Act 2010, and with ethical approval from the University of St Andrews Animal Welfare and Ethics Committee.

Briefly, seals are captured in seine, tangle, pop-up, or hand-held nets when hauled out on land. Animals are held in hoop nets, and, in recent years, sedated with 10 mg/100 kg of intramuscular Midazolam (Dechra, Oudewater, The Netherlands), then anaesthetised with either Zoletil (Virbac, Caros, France) at an intravenous (i.v.) dose rate of 0.5 mg/100 kg, or Medetomidine hydrochloride (Dechra, Oudewater, The Netherlands, 2.5 mg/100 kg i.v.) and Butorphanol (Zoetis UK Ltd., Leatherhead, UK, 10 mg/100 kg i.v.). The Medetomidine was reversed using Atipamezole hydrochloride (Forte healthcare, Dublin, Ireland, 12.5 mg/100 kg i.v.). Blood samples were collected from the extradural vein into plain Vacutainer tubes (Becton Dickinson, Oxford UK), centrifuged at 1000× *g* for 10 min as soon as possible post-collection, and the extracted serum sent within 24 h for analysis or stored at −20 °C until analysis. Haematology was not included because, where samples could not be sent within 24 h due to the remoteness and logistics of the field sites, analysis could not be carried out on stored samples. The reduced sample size was then too small for robust analysis.

Animals are categorised into age classes based on standard nose-tail length, and those >110 cm were classed as adults [34] and included in this dataset. Samples from subadults were excluded due to potential age-specific differences in some parameters [5] and a small sample size. Where only mass was available (*n* = 14), nose-tail length was estimated from the statistically significant relationship between mass and length, using the data from all the adults by sex (see Supplementary Materials Figure S1). All were >50 kg in mass and therefore >110 cm in length.

**Figure 1:**
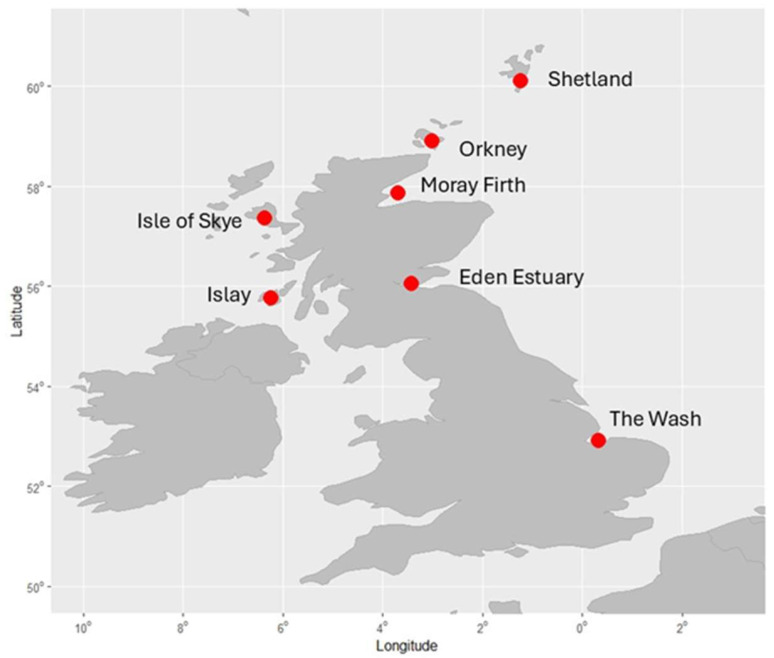
Map of the UK showing the locations of the harbour seal capture and release haulout sites.

Details of the samples collected by location (Figure 1) and year are shown in Table 1. Seals were captured throughout the year, and many of the females would have been pregnant at the time of sampling, given the high pregnancy rate among harbour seals [41]. The samples spanning the longest period and contributing the most to the dataset were from Moray Firth (Table 1), with similar numbers of males (*n* = 166) and females (*n* = 151) being sampled.

### 2.2. Sample Analysis

Overall, up to 251 samples from adult harbour seals were included in the clinical chemistry parameter analysis (with some subsets being analysed for additional parameters resulting in uneven sample sizes between *n* = 213 and *n* = 251), and 271 were analysed for total triiodothyronine (TT3) which has been found to be important in understanding the health of harbour seals and bottlenose dolphins [23,40]. All samples were sent to the same IDEXX Veterinary Laboratory (IDEXX Veterinary Laboratories, Wetherby, UK) for the analysis of the complete chemistry panel for seals by automated biochemical analyser, thus eliminating any potential inter-laboratory variation. All biochemical tests (*n* = 19 parameters, Table 2) were carried out for the samples from all regions except Shetland and the Eden Estuary, and a small number of samples from the Moray Firth, where a subset of the parameters were reported (Table 2). TT3 was measured using a commercial ELISA (Fortress Diagnostics, Belfast, UK) that has been previously verified for seal serum [40]. The mean inter-assay CV (10 samples across 4 plates) was 14.86%, and the mean intra-assay CV (10 samples within a plate) was 7.6%. Samples for TT3 from all regions were analysed at the Sea Mammal Research Unit, University of St Andrews, shortly after collection, between 1998 and 2025.

### 2.3. Clinical Chemistry Reference Intervals Generation

All data analyses were conducted in R [42]. Reference intervals were generated using the *refineR* package (version 2.0.0) with bootstrap resampling (*n* = 200) and 90% confidence intervals around the 2.5th and 97.5th percentiles. *RefineR* (version 2.0.0) [38] is a package that runs within the R programme [43,44], and builds on similar approaches to indirect reference interval generation using RWD (e.g., Reference Interval Estimation from Mixed Distributions using Truncation Points and the Kolmogorov–Smirnov Distance, (kosmic method [45])). *RefineR* assumes that the distribution of the non-pathological samples can be modelled with a Box–Cox transformed normal distribution and, using an inverse modelling approach, finds the model that can best explain the observed data. Where distributions were skewed, and the shift parameter was not equal to zero, a 2-parameter Box–Cox transformation was fitted [44]. The model parameters and the transformation used for each of the analytes are given in the Supplementary Materials (Table S1). For details of the methodology and results of a performance evaluation against other RWD approaches, see Refs [38,44].

Given the sample size requirements for robust reference intervals and the uneven sampling distribution by region (Table 1), differences between the sexes were not assessed.

## 3. Results

**Figure 2:**
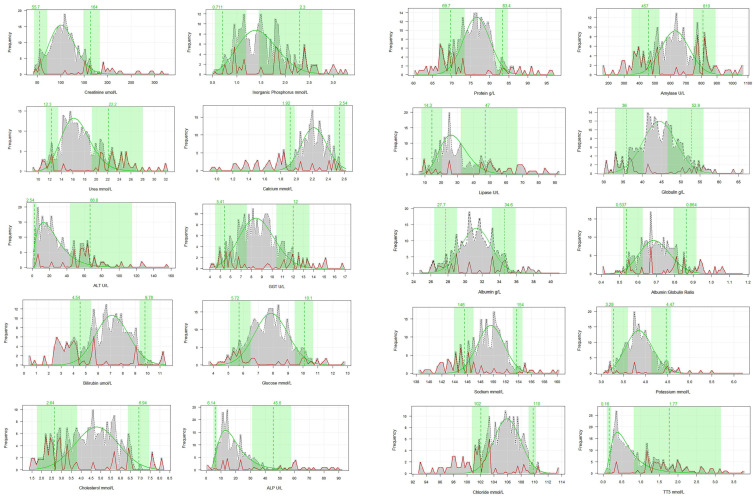
The reference interval point estimates (dashed vertical lines) with their 90% CIs (green shaded area) and distribution by clinical chemistry parameter. CI Proportion = 90%; point estimate = Median, bootstrapped; pathological distribution = red continuous line.

The resulting reference interval point estimates (median bootstrapped values) with their 90% confidence intervals (CIs) and sample sizes are given in Table 3, and the model parameters from the analyses for each analyte are given in the Supplementary Materials (Table S1). The distribution plots, reference intervals, and confidence bounds are also shown in Figure 2. The minimum and maximum values for each analyte can be found in the Supplementary Materials (Table S2). There was considerable variability among the parameters in the width of both the upper and lower 90% CI. Notably, both limits for some were very wide (e.g., phosphorus and cholesterol) due to the high degree of individual variability, whereas others (e.g., creatinine, sodium, and glucose) showed a much stronger central tendency. The distributions of many were, as expected, skewed either positively (e.g., alanine transaminase) or negatively (e.g., calcium). In many of these, one limit was considerably wider than the other (e.g., total triiodothyronine and alanine transaminase). This asymmetry is due to underlying demographic factors and disease, highlighting the importance of using a robust statistical approach [38].

## 4. Discussion

This study presents comprehensive and robust reference intervals for free-living UK harbour seals. These can be used diagnostically in animals under veterinary care, during rescue and rehabilitation, and for the wider understanding of population health, where the proportion of individuals with abnormal results can be evaluated to understand the degree of impairment within a population [27].

Previous work aiming to establish both clinical chemistry and haematological reference intervals for harbour seals, and indeed pinnipeds more generally, has been heavily biased towards rescued, rehabilitated, and captive animals [46,47,48,49]. Typically, these data can then be used to indicate clinical changes in seals undergoing rehabilitation and provide an objective measure of when individuals may be eligible for release back into the wild [49]. However, the relative representativeness of these ranges when applied to wild seals that have never been either housed in managed care or in rehabilitation facilities is likely limited. Only a handful of previous studies have reported reference intervals for wild harbour seals, but these have been limited by smaller sample sizes [37,50], and/or a focus on pups and juveniles [37,51]. Thus, there has previously been a lack of comprehensive, comparable clinical chemistry data with which to establish reference intervals, specifically for adults of this species. In terms of understanding population trajectories, from both a management and a conservation perspective, adult health and survival are key parameters for modelling population growth rates. As such, information to better interpret physiological parameters linked to individual health and subsequent survival probability is imperative to predict population trends and drivers of changes in abundance [26].

The application of the *refineR* algorithm to wildlife data is novel and entirely suitable for this application, where pathological as well as non-pathological samples are included. However, sample sizes still need to be relatively large (*n* > 100) for the models to fit the data [38]. There will also still be areas of uncertainty, some of which are captured in the 90% confidence intervals; nevertheless, health and risk assessments should be made with the degree of uncertainty in mind. Conclusions at different levels will undoubtedly be made depending on the situation, and a cautionary approach would be advisable, the degree of which is likely to differ for veterinary decision making compared to a population-level risk assessment, for example [12].

This study reports a single reference interval for each parameter and does not investigate differences by sex. There is widespread discussion in the literature regarding the criteria for subgroup portioning and when and where this is necessary [52]. Indeed, other studies have shown that the majority of analytes (excluding hormones and some analytes that are related to muscle mass) did not warrant separate reference intervals, a finding also reported in marine mammals [21]. Greig et al. [37] investigated sex differences in wild adult harbour seals and found in a panel of 22 analytes that sex was retained in a predictive model only for blood urea nitrogen. Here, we have a relatively even sample size of males and females, so the reference intervals will represent a combination of the sexes rather than a biased one. The problem is also diminished because this dataset is restricted to adults, and it is among juveniles that are undergoing rapid physiological changes during growth and development that most differences are likely to be observed [53]. Harris and Boyd [54] provided some guidelines for deciding whether separate ranges should be employed. Specifically, the proportions of distributions calculated when the data are divided into subgroups that fall outside the combined reference limits should be kept close to 2.5%. However, investigating differences by region as well as sex will require larger sample sizes [55], and the aim is to address this in future studies.

Of importance is how the parameters are used to diagnose health or disease. The significance of the directionality is critical, as values below the reference interval may indicate a different disease process compared to a diagnosis when the observed value is above the reference interval. For example, high phosphate levels (hyperphosphataemia) could, among other things, indicate renal failure, hypoparathyroidism, or vitamin D intoxication, whereas low phosphate levels (hypophosphataemia) might be a sign of vitamin D deficiency or hyperparathyroidism. As such, the shape of the distribution of the parameters and the nature of the blood chemistry, how much it can vary within an individual animal, and the influence of preanalytical variables, such as diet, time of day, and season will impact the width of the reference interval and the size of the confidence intervals around the upper and lower limits [56]. For example, the width of the upper and lower confidence intervals for phosphorus and cholesterol seen here is very wide compared to the electrolytes, which are under much tighter physiological control (Supplementary Materials Figure S2). Thus, the causes of biological variability are likely to be a combination of the factors listed above, in addition to diurnal and other intrinsic variation, sex, genetic variation, reproductive status, age, habitat, location, and population heterogeneity are all likely to be responsible to varying extents [5,6,21]. Extrinsic factors may also be involved, including time for analysis. This highlights the importance of a sufficiently large sample size such that the “true” reference interval will lie between the confidence limits. The higher degrees of variability observed in certain parameters, particularly in the upper limits for the liver enzymes, were also seen in reference intervals published for other adult marine mammal species, including phocid seals [6,21,37,50].

Although individual parameters are often evaluated separately and can be highly indicative of perturbations, it is often changes in a combination of parameters that are diagnostically meaningful (as grouped in Table 1), particularly at the individual level [56], but also at the population level. This is especially the case for many disease conditions that are multi-organ, affecting a number of different systems simultaneously. In the expert-based system to predict population survival rates from health data in a long-term study of photo-identified bottlenose dolphins (VESOP or Veterinary Expert System for Outcome Prediction model), the panel assessed the diagnostic value of a suite of clinical chemistry tests and was able to identify 11 critical measures that were indeed important in predicting survivorship [2]. Whilst many of the measures were individually important, it was the combination of abnormalities that was the most informative, with more predictive power. These core measures, notably combinations of inflammatory markers (globulins, albumins, and ALP), in conjunction with the reference intervals reported here, might then also be used to investigate survival probability in harbour seals. Together with haematology and morphometric data, they may be subsequently used in health assessments and predictive models.

The debate regarding the definition of health for wildlife is a complex one that has resulted in a discussion that highlights that normal health status is much more than the absence of disease [57]. It has been argued that the definition of wildlife health should include more than the view that it is a state where an organism “does not experience drastic changes in physical appearance or normal functions” [58]. Hanisch et al. [59] carried out an expert elicitation exercise, which concluded that wildlife health, largely assessed at the population rather than the individual level, should include concepts such as the resilience of the population and its sustainability. One way to measure this is to employ a combined assessment of population dynamics, growth, and health metrics in which clinical chemistry and other health parameters, such as body condition and its relationship to survival probability, play a central role.

## 5. Conclusions

As integrators of exposure to multiple stressors, health metrics that can be obtained from capture–release studies in wildlife are gaining acceptance as central to improving our understanding of wider impacts. Thus, baseline reference information, such as is reported here, is increasingly important for wildlife health studies and becomes critical when unpredicted environmental impacts, such as major oil spills, affect wildlife health. As the importance of assessing the impacts of anthropogenic activities on marine wildlife increases, baseline health data to compare against ongoing monitoring initiatives forms a key part of predicting population-level effects to advise conservation, policy, and management decisions.

## Figures and Tables

**Table 1:** Location and years of blood sample collection for male and female adult UK harbour seals.

Location	Time Period	Males	Females	Number of Animals
Shetland	2022	8	21	29
Orkney	2016–2017	13	21	34
Moray Firth	2008–2025	63	56	119
Eden Estuary	1998–2000	33	33	66
The Wash	2023	22	15	37
Islay	2024	17	1	18
Isle of Skye	2017	10	4	14
Total		166	151	317

**Table 2:** Biochemical parameters analysed in harbour seal serum samples.

Function	Parameter
Renal	Creatinine (µmol/L)
	Phosphorous (mmol/L)
	Urea (mmol/L)
	Calcium (mmol/L)
Hepatic	Alanine Transaminase (ALT) (U/L)
	Gamma-glutamyl Transferase (GGT) (U/L)
	Bilirubin (µmol/L)
Nutritional status/ gastrointestinal	Glucose (mmol/L)
	Cholesterol (mmol/L)
	Alkaline Phosphatase (ALP) (U/L)
	Total protein (g/L)
	Amylase (U/L)
	Lipase (U/L)
Infection/inflammation	Globulin (g/L)
	Albumin (g/L)
Electrolytes	Sodium (mmol/L)
	Potassium (mmol/L)
	Chloride (mmol/L)
Thyroid	Triiodothyronine (nmol/L)

**Table 3:** Reference interval thresholds (2.5th and 97.5th percentiles) and associated 90% confidence intervals (CIs) of clinical chemistry parameters generated for adult UK harbour seals.

Parameter	Sample Size	Lower 2.5th Percentile	Lower 90% CI	Upper 97.5th Percentile	Upper 90% CI
Creatinine µmol/L	249	55.74	43.50–71.32	163.73	148.70–183.59
Phosphorus mmol/L	218	0.711	0.543–1.198	2.30	1.46–2.77
Urea mmol/L	251	12.29	11.38–13.49	22.18	19.27–28.03
Calcium mmol/L	218	1.92	1.87–1.96	2.54	2.50–2.66
Alanine Transaminase (ALT) U/L	251	2.54	1.00–5.00	66.85	43.61–115.07
Gamma-glutamyl Transferase (GGT) U/L	222	5.41	4.52–7.57	12.04	10.41–13.61
Bilirubin µmol/L	242	4.54	3.73–5.44	9.78	9.34–10.34
Glucose mmol/L	242	5.72	5.13–6.50	10.12	9.46–10.69
Cholesterol mmol/L	218	2.64	1.76–3.79	6.94	6.37–7.46
Alkaline Phosphatase (ALP) U/L	248	6.14	4.89–7.43	45.59	31.24–57.89
Total Protein g/L	251	69.71	66.70–73.39	83.37	81.44–84.76
Amylase U/L	213	457.00	349.62–529.04	809.79	747.65–891.63
Lipase U/L	213	14.33	7.89–20.57	47.00	32.01–67.00
Globulin g/L	251	35.97	32.93–40.35	52.86	46.55–55.88
Albumin g/L	251	27.73	26.41–29.06	34.62	33.12–35.92
Albumin: Globulin ratio	251	0.537	0.518–0.626	0.864	0.793–0.918
Sodium mmol/L	221	145.57	143.90–146.94	153.69	153.10–154.60
Potassium mmol/L	221	3.28	3.23–3.61	4.47	4.13–4.57
Chloride mmol/L	219	102.10	100.75–103.23	109.80	109.14–110.22
Total Triiodothyronine nmol/L	271	0.160	0.954–0.212	1.770	0.822–3.160

## Data Availability

The data used in this analysis is available from figshare https://doi.org/10.6084/m9.figshare.30416344 (accessed on 24 November 2025).

## Supplementary Materials

The following supporting information can be downloaded at: https://www.mdpi.com/article/10.3390/ani15233429/s1, Figure S1: Mass (log10 transformed) to length relationships for male and female harbour seals. Males: length = log10(mass) ∗ 85.961 − 19.311, *p* < 0.0001, R^2^ = 0.7492. Females: length = log10(mass) ∗ 66.806 + 12.916, *p* < 0.0001, R^2^ = 0.6299. Table S1: *RefineR* Fitted Model Parameters by analyte. Table S2: Minimum and Maximum values for clinical chemistry parameters measured in wild, adult harbour seals.

## Abbreviations

The following abbreviations are used in this manuscript:

IFCC	International Federation of Clinical Chemistry
RWD	Real-World Data
TT3	Total Triiodothyronine
i.v.	Intravenous
GGT	Gamma-glutamyl Transferase
ALP	Alkaline Phosphatase
ALT	Alanine Transaminase
CI	Confidence Interval
VESOP	Veterinary Expert System for Outcome Prediction
