# Supplementary material for: Clinical Chemistry Reference Intervals for Health Assessment in Wild Adult Harbour Seals
Source: Animals (Basel). 2025 Nov 27;15(23):3429. doi: 10.3390/ani15233429 (PMC12691109; doi:10.3390/ani15233429)
Supplement: Supplementary file 1 [file animals-15-03429-s001.zip › animals-3973907-supplementary.pdf]

Supplementary Materials

Mass (log10 transformed) to length relationships were used to estimate length for missing data (14 animals with missing length measurements). Males and females were treated separately and using their mass all were estimated to be >110 cm, and therefore adults.

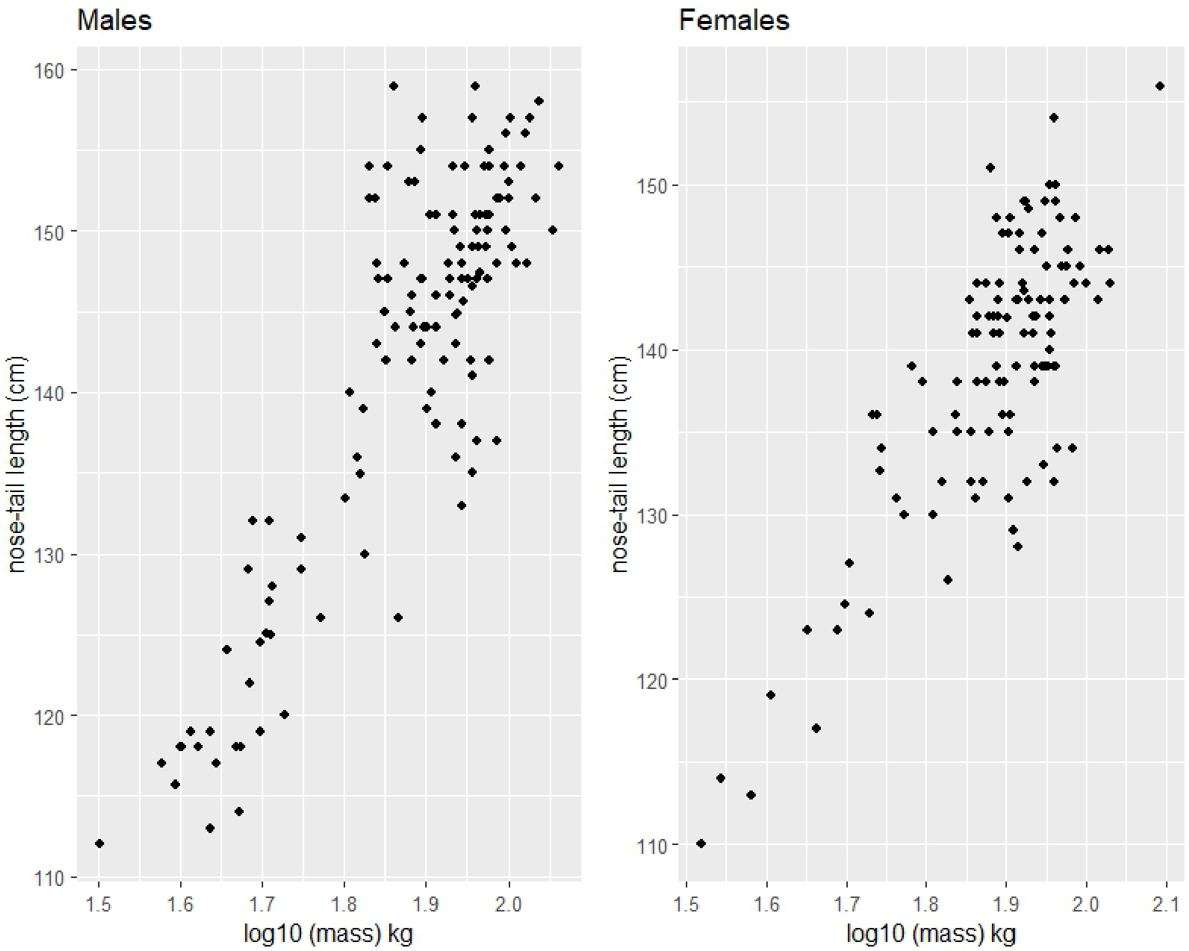

Figure. S1. Mass (log10 transformed) to length relationships for male and female harbour seals. Males: length = log10(mass) * 85.961 – 19.311, $p<0.0001$, $R^2 = 0.7492$. Females: length = log10(mass) * 66.806 + 12.916, $p<0.0001$, $R^2 = 0.6299$.

Table S1. *RefineR* Fitted Model Parameters by analyte

Creatinine	Phosphorus	Urea	Calcium	Alanine Transaminase (ALT)	Gamma-glutamyl Transferase (GGT)
model: BoxCox	model: modBoxCox	model: modBoxCox (2-parameter)	model: BoxCox	model: modBoxCox (2-parameter)	model: BoxCox
N data: 249	N data: 218	N data: 251	N data: 218	N data: 251	N data: 222
N bootstrap: 200	N bootstrap: 200	N bootstrap: 200	N bootstrap: 200	N bootstrap: 200	N bootstrap: 200
lambda: 1	lambda: 0.89	lambda: 0.00274	lambda: 0.593	lambda: 0.122	lambda: 0.507
mu: 102	mu: 0.151	mu: 2.08	mu: 1.02	mu: 3.86	mu: 4.03
sigma: 27.9	sigma: 0.439	sigma: 0.451	sigma: 0.114	sigma: 1.22	sigma: 0.501
shift: 0	shift: 0.3	shift: 9.06	shift: 0	shift: 0.198	shift: 0
cost: -11.7	cost: -6.75	cost: -9.07	cost: -9.11	cost: -7.57	cost: -6.69
NP fraction: 0.863	NP fraction: 0.829	NP fraction: 0.953	NP fraction: 0.717	NP fraction: 0.883	NP fraction: 0.71

Bilirubin	Glucose	Cholesterol	Alkaline Phosphatase ALP	Total Protein	Amylase
model: BoxCox	model: BoxCox	model: BoxCox	model: modBoxCox (2-parameter)	model: BoxCox	model: BoxCox
N data: 242	N data: 242	N data: 218	N data: 248	N data: 251	N data: 213
N bootstrap: 200	N bootstrap: 200	N bootstrap: 200	N bootstrap: 200	N bootstrap: 200	N bootstrap: 200
lambda: 0.786	lambda: 0.0109	lambda: 0.963	lambda: 0.00274	lambda: 1	lambda: 1
mu: 4.79	mu: 2.1	mu: 3.72	mu: 2.75	mu: 75	mu: 616
sigma: 0.799	sigma: 0.136	sigma: 1.15	sigma: 0.626	sigma: 4.06	sigma: 133
shift: 0	shift: 0	shift: 0	shift: 1.62	shift: 0	shift: 0
cost: -5.94	cost: -5.91	cost: -8.76	cost: -8.21	cost: -7.68	cost: -9.29
NP fraction: 0.648	NP fraction: 0.76	NP fraction: 0.818	NP fraction: 0.856	NP fraction: 0.86	NP fraction: 0.843

Lipase	Globulin	Albumin	Albumin:Globulin Ratio	Sodium	Potassium	Chloride
model: BoxCox<br>N data: 213<br>N bootstrap: 200<br>lambda: 0.89<br>mu: 19<br>sigma: 2.61<br>shift: 0<br>cost: -7.39<br>NP fraction: 0.585	model: BoxCox<br>N data: 251<br>N bootstrap: 200<br>lambda: 0.0637<br>mu: 4.29<br>sigma: 0.148<br>shift: 0<br>cost: -8.66<br>NP fraction: 0.942	model: BoxCox<br>N data: 251<br>N bootstrap: 200<br>lambda: 0.854<br>mu: 20.9<br>sigma: 1.36<br>shift: 0<br>cost: -7.69<br>NP fraction: 0.968	model: BoxCox<br>N data: 244<br>N bootstrap: 200<br>lambda: 0.244<br>mu: -0.341<br>sigma: 0.13<br>shift: 0<br>cost: -5.66<br>NP fraction: 0.929	model: BoxCox<br>N data: 221<br>N bootstrap: 200<br>lambda: 0.927<br>mu: 111<br>sigma: 1.42<br>shift: 0<br>cost: -7.33<br>NP fraction: 0.744	model: BoxCox<br>N data: 221<br>N bootstrap: 200<br>lambda: 0.244<br>mu: 1.6<br>sigma: 0.112<br>shift: 0<br>cost: -11.5<br>NP fraction: 0.952	model: BoxCox<br>N data: 219<br>N bootstrap: 200<br>lambda: 0.854<br>mu: 61.6<br>sigma: 1.01<br>shift: 0<br>cost: -8.4<br>NP fraction: 0.738

Total Triiodothyronine
model: modBoxCox (2-parameter)<br>N data: 271<br>N bootstrap: 200<br>lambda: 0.0595<br>mu: -0.695<br>sigma: 0.669<br>shift: 0.056<br>cost: -6.62<br>NP fraction: 0.734

Table S2. Minimum and Maximum values for clinical chemistry parameters measured in wild, adult harbour seals

Parameter	Minimum	Maximum
Creatinine (µmol/L)	45	400
Phosphorous (mmol/L)	0.55	5.11
Urea (mmol/L)	8.8	121.3
Calcium (mmol/L)	0.07	2.55
Alanine Transaminase (ALT) (U/L)	0.8	195.2
Gamma-glutamyl Transferase (GGT) (U/L)	3.7	43
Bilirubin (µmol/L)	0.4	12.6
Glucose	2.6	13.4
Cholesterol (mmol/L)	1.0	8.8
Alkaline Phosphatase (ALP) (U/L)	0.1	25.3
Total Protein (g/L)	56	100
Amylase (U/L)	166.0	1068.6
Lipase (U/L)	6.2	951.6
Globulin (g/L)	28.1	71.1
Albumin (g/L)	23.5	43.6
A:G Ratio	0.41	1.42
Sodium (mmol/L)	131.0	160.5
Potassium (mmol/L)	3.09	10
Chloride (mmol/L)	92.6	115.0
Triiodothyronine (nmol/L)	0.11	7.87
